# The triple combination of tenofovir, emtricitabine and efavirenz shows synergistic anti-HIV-1 activity *in vitro*: a mechanism of action study

**DOI:** 10.1186/1742-4690-6-44

**Published:** 2009-05-13

**Authors:** Joy Y Feng, John K Ly, Florence Myrick, Derrick Goodman, Kirsten L White, Evguenia S Svarovskaia, Katyna Borroto-Esoda, Michael D Miller

**Affiliations:** 1 Gilead Sciences, Inc, 333 Lakeside Drive, Foster City, California, 94404, USA

## Abstract

**Background:**

Tenofovir disoproxil fumarate (TDF), emtricitabine (FTC), and efavirenz (EFV) are the three components of the once-daily, single tablet regimen (Atripla) for treatment of HIV-1 infection. Previous cell culture studies have demonstrated that the double combination of tenofovir (TFV), the parent drug of TDF, and FTC were additive to synergistic in their anti-HIV activity, which correlated with increased levels of intracellular phosphorylation of both compounds.

**Results:**

In this study, we demonstrated the combinations of TFV+FTC, TFV+EFV, FTC+EFV, and TFV+FTC+EFV synergistically inhibit HIV replication in cell culture and synergistically inhibit HIV-1 reverse transcriptase (RT) catalyzed DNA synthesis in biochemical assays. Several different methods were applied to define synergy including median-effect analysis, MacSynergy^®^II and quantitative isobologram analysis. We demonstrated that the enhanced formation of dead-end complexes (DEC) by HIV-1 RT and TFV-terminated DNA in the presence of FTC-triphosphate (TP) could contribute to the synergy observed for the combination of TFV+FTC, possibly through reduced terminal NRTI excision. Furthermore, we showed that EFV facilitated efficient formation of stable, DEC-like complexes by TFV- or FTC-monophosphate (MP)-terminated DNA and this can contribute to the synergistic inhibition of HIV-1 RT by TFV-diphosphate (DP)+EFV and FTC-TP+EFV combinations.

**Conclusion:**

This study demonstrated a clear correlation between the synergistic antiviral activities of TFV+FTC, TFV+EFV, FTC+EFV, and TFV+FTC+EFV combinations and synergistic HIV-1 RT inhibition at the enzymatic level. We propose the molecular mechanisms for the TFV+FTC+EFV synergy to be a combination of increased levels of the active metabolites TFV-DP and FTC-TP and enhanced DEC formation by a chain-terminated DNA and HIV-1 RT in the presence of the second and the third drug in the combination. This study furthers the understanding of the longstanding observations of synergistic anti-HIV-1 effects of many NRTI+NNRTI and certain NRTI+NRTI combinations in cell culture, and provides biochemical evidence that combinations of anti-HIV agents can increase the intracellular drug efficacy, without increasing the extracellular drug concentrations.

## Background

Combination of anti-HIV agents has long been an indispensable tool in fighting the AIDS epidemic. Combination of drugs from different classes has proven to be beneficial in terms of sustained efficacy and long-term safety, provided there are no significant negative pharmacokinetic drug-drug interactions. Among all of the anti-HIV drugs in development or in the clinic, combinations of nucleoside or nucleotide reverse transcriptase (RT) inhibitor (NRTI) and non-nucleoside RT inhibitor (NNRTI) have been the most extensively studied. NRTI are transformed into their active tri- or diphosphate (TP or DP) forms by cellular kinases [1]. Structurally resembling the natural dNTPs, the active metabolites of NRTIs serve as alternative substrates for HIV-1 RT during viral DNA synthesis, which results in chain-termination of DNA elongation due to the lack of the 3'-hydroxy moiety. The incorporated NRTIs can be removed, however, by pyrophosphate- (PP_i_) or ATP-mediated excision that occurs at a basal level for wild-type RT and can be accelerated or diminished by different RT mutations, such as thymidine analog mutations or K65R, respectively [2-4]. NNRTI inhibit HIV-1 replication through multiple mechanisms [5], but mainly by inducing conformational changes within HIV-1 RT at the polymerase active site which significantly slow down viral DNA synthesis but have no effect on the binding affinity of natural dNTP and primer/template [6].

Many NRTI+NNRTI combinations show synergistic anti-HIV activities in cell culture [7-12]. Synergistic effects were also shown by drug combinations in HIV-1 RT enzymatic assays [12-15]. The enhanced potency of the AZT+NVP combination in comparison to AZT alone was reported in a clinical trial study [16]. Two major mechanisms of synergy have been proposed: (1) NNRTI inhibited the PPi- or ATP-mediated removal of zidovudine (AZT)-monophosphate (MP) from the 3'-end of the DNA primer [17-20]; and (2) NNRTI accelerated HIV-1 RT's RNase H activity and thus diminished NRTI excision [21].

Interest in the NRTI+NRTI combinations was first ignited during the HIV monotherapy era by the surprisingly synergistic effects of AZT+ddI both *in vitro *and in clinical trial studies [22-24], in the absence of a pharmacokinetic interaction between the two drugs [25]. Additional *in vitro *NRTI combination studies showed synergistic antiviral activity in cell culture, including (but not limited to) AZT + either carbovir (CBV, the metabolite of abacavir (ABC)), ddC, 3TC, FTC, or TFV [26-29], TFV+ddI [29], and TFV+FTC [30]. To our knowledge, TFV+FTC synergy was the only one that has been correlated with statistically significant increases in the levels of the active metabolites [30]. Most recently, a study on anti-HIV-1 synergy of a panel of NRTI+NRTI combinations in peripheral blood mononuclear cells (PBMC) claimed antagonistic effect of TFV+ABC [31], contradicting an earlier report on the additive antiviral effect TFV+ABC tested in the same cell line.[32]

The biochemical studies on the above mentioned synergistic NRTI combinations have been somewhat controversial, likely due to various experimental designs and different methods of analysis. For example, using defined sequences of RNA or DNA templates, White *et al*. reported combinations of AZT-TP with ddCTP, ddATP, or CBV-TP to be additive [33]. Also using a template with defined sequence, Villahermosa *et al*. reported that the combination of AZT-TP and ddCTP was merely additive under conventional conditions where the template:primer was in large excess over the enzyme concentration; however, when the enzyme was in large excess over the template:primer, the combined inhibition effects of AZT-TP and ddCTP were synergistic [34]. Periclou and colleagues reported combinations of AZT-TP+ddATP and AZT-TP+ddATP+3TC-TP synergistically inhibited HIV-1 RT, based on a mathematic model in which the rate of DNA synthesis was determined using the four natural dNTP substrates and their competitive NRTI analogs [25].

There are many methods available to analyze the effect of drug combinations [35-37]. Synergy and antagonism are commonly defined as a greater or lesser pharmacological effect than would be predicted for an additive effect. Mathematically, there are two major definitions of additivity: Bliss Independence and Loewe Additivity. Bliss Independence states that additivity occurs when two agents act independently of the other. Loewe Additivity defines the effects seen with a second drug present are the same as that seen when a drug is added to itself; in other words, when a drug is tested in combination with itself, the observed effect is defined as additive. Among the many frequently used methods, the median-effect method by Chou and Talalay [38,39], the isobologram analysis [40,41], and the Berebaum combination indices [35] are based on Loewe Additivity, while the MacSynergy II analysis [42] is based on Bliss Independence. All of these four methods are accompanied with statistical analyses. The Yonetani-Theorell Plot was first developed as a simple graphical method to quantify the interaction of two competitive inhibitors acting on the same enzyme [43] and was later adopted to study drug combinations.

The combination of TDF, FTC, and EFV makes up the components of the once-daily single tablet regimen (Atripla) for treatment of HIV-1 infection [44]. In this paper, we studied the drug combinations TFV+FTC, TFV+EFV, FTC+EFV, and TFV+FTC+EFV in both cell-based assays and HIV-1 RT enzymatic assays. We used different methods to analyze the effects of the combinations to minimize bias associated with a specific method. Furthermore, we demonstrated that HIV-1 RT and TFV-terminated DNA form dead-end complex (DEC) in the presence of FTC-TP, which could contribute to the synergistic inhibition of HIV-1 RT by TFV-DP+FTC-TP at the enzymatic level. Our data also showed that EFV greatly facilitates the formation of stable, DEC-like complexes with HIV-1 RT and TFV- or FTC-MP-terminated DNA.

## Results

### Two- and three-drug combinations of TFV, FTC, and EFV showed synergistic anti-HIV activity in cell culture

**Figure 1:**
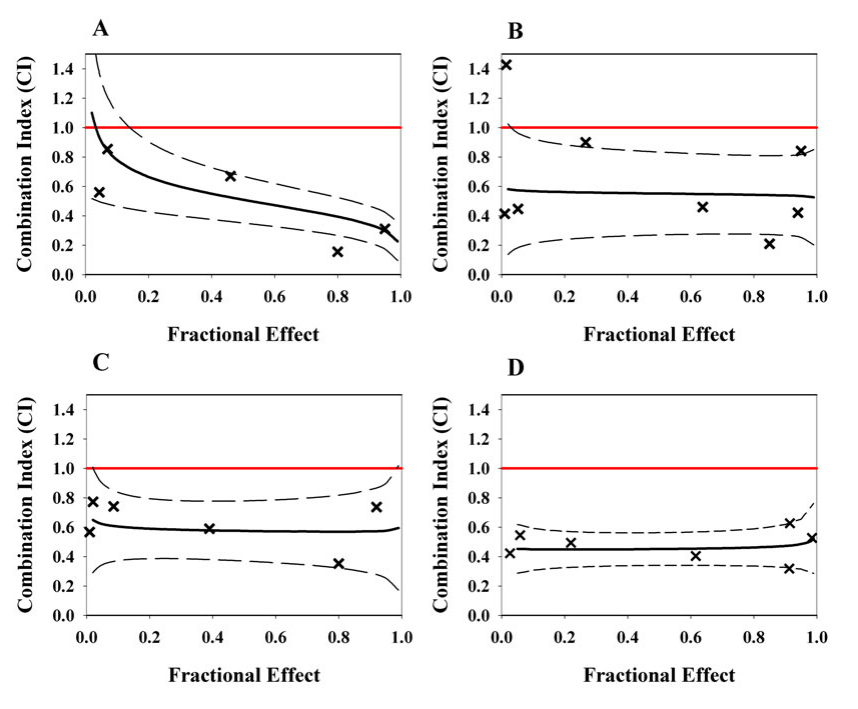
**Synergistic inhibition of HIV-1 replication by combinations TFV+FTC, TFV+EFV, FTC+EFV, and TFV+FTC+EFV analyzed by the median-effect analysis**. The solid line presents curve fitting of the CI values as a function of fractional effect. The dashed lines represent 95% confidence interval. The line (in red) at CI = 1 represents additivity. (A) TFV+FTC (1:1 IC_50 _ratio) with an average CI of 0.47; (B) TFV+EFV (1:1 IC_50 _ratio) with average CI of 0.54; (C) FTC+EFV (1:1 IC_50 _ratio) with an average CI of 0.62; (D) TFV+FTC+EFV (1:1:1 IC_50 _ratio) with an average CI of 0.46.

TFV, FTC, and EFV were tested in two-drug and three-drug combinations for antiviral activity against HIV-1 in MT-2 cells. The EC_50 _value for each single drug was 13 μM, 1.3 μM, and 5.6 nM for TFV, FTC and EFV, respectively. For the median-effect analysis, combinations of TFV+FTC, TFV+EFV, FTC+EFV, and TFV+FTC+EFV were synergistic, as shown by the representative curves in Fig. 1 (panels A to D) with calculated combination index (CI) values below the additivity line (CI = 1), and with CI values of 0.52, 0.51, 0.59, and 0.56, respectively (Table 1).

**Table 1:** Evaluation of drug combinations for inhibition of HIV-1 in MT-2 cell culture.

Combinations	Analysis Method		
	
	Median-Effect^a^ (Combination Index)	MacSynergy^b^ (Synergy/Antagonism Volumes μM^2^%)	Isobologram^c^ (ADA, *p *value)
TFV+FTC	synergy (0.52 ± 0.08)	strong synergy (181 ± 30/-36 ± 10)	synergy (-0.37, 0.001)
TFV+EFV	synergy (0.51 ± 0.14)	strong synergy (267 ± 50/-13 ± 5)	synergy (-0.14, 0.027)
FTC+EFV	synergy (0.59 ± 0.11)	moderate synergy (90 ± 30/0 ± 10)	synergy (-0.23, 0.001)
TFV+FTC+EFV	synergy (0.56 ± 0.12)	ND^d^	ND

^a ^The drugs were mixed at 1:1 IC_50 _ratios. Definition of the degrees of synergy is described in Methods. The values are averages of more than three independent measurements.

^b ^Synergy/antagonism volumes were calculated at the 95% confidence level. Definition of the degrees of synergy is described in Methods.

^c ^Synergy is defined by a negative ADA (the average deviation from dose-wise additivity) value with *p *value ≤ 0.05.

^d^ND = not determined.

**Figure 2:**
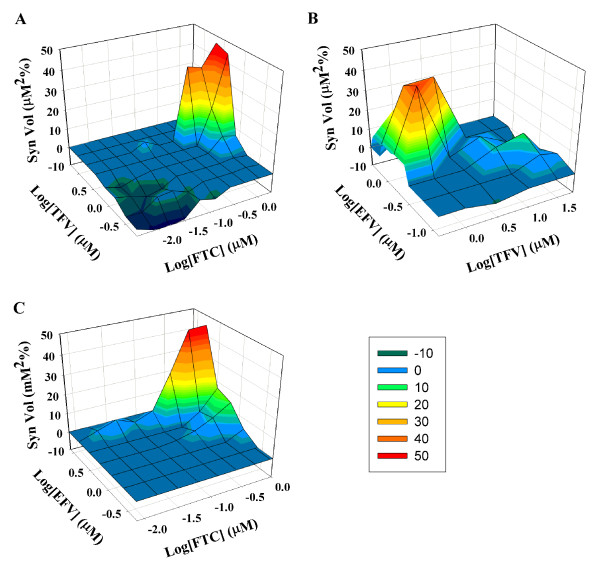
**Synergistic inhibition of HIV-1 replication by combinations of (A) TFV+FTC, (B) TFV+EFV, and (C) FTC+EFV analyzed by MacSynegy II**. Calculated additive antiviral interactions were subtracted from experimentally determined values to reveal regions and corresponding concentrations at which synergistic antiviral interactions occurred. Peaks of statistically significant (95% confidence level) synergy are shown in colors from dark blue to red, with red indicating the strongest synergy. Values used to describe the percentage of inhibition above the expected were derived from five experiments.

For the MacSynergy analysis, combinations of TFV+FTC, TFV+EFV, and FTC+EFV were strongly synergistic, as indicated by the high peak of synergy above the flat plane of additivity (Fig. 2A and 2B) and overall synergy volumes of 181 μM^2^% and 267 μM^2^%, respectively (Table 1). The combination of FTC+EFV was moderately synergistic (Fig 1C) with a synergy volumes of 90 μM^2^% (Table 1).

**Figure 3:**
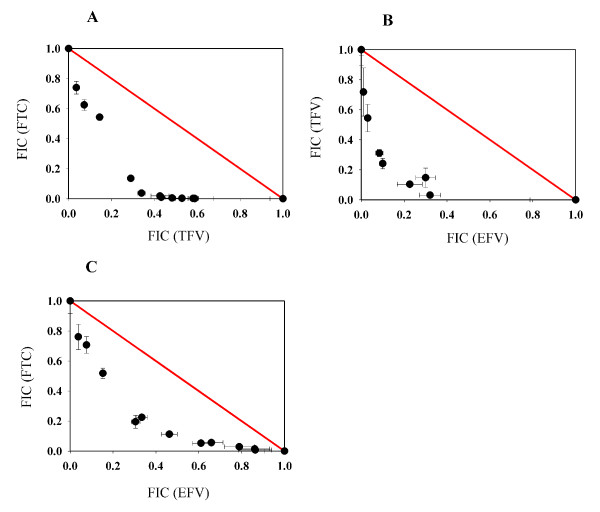
**Synergistic inhibition of HIV-1 replication by drug combinations analyzed by the isobologram analysis**. (A) The combination of TFV+FTC with an ADA value of -0.37 (*p *< 0.001); (B) The combination of TFV+EFV with an ADA value of -0.14 (*p *< 0.03); (C) The combination of FTC+EFV with an ADA value of -0.23 (*p *< 0.001). The diagonally drawn solid line (in red) represents additivity.

For the isobologram analysis, the combinations of TFV+FTC, TFV+EFV, and FTC+EFV are shown in Fig. 3 (panel A to C), where experimental data points are below the calculated additivity line indicating synergistic effects of the combinations. As summarized in Table 1, the combinations of TFV+FTC, TFV+EFV, and FTC+EFV were synergistic with ADA values of -0.37 (*p *= 0.001), -0.14 (*p *= 0.027), and -0.23 (*p *= 0.001), respectively. Overall, all of the combinations of TFV, FTC, and EFV showed synergy, and none of the combinations was antagonistic.

### TFV-DP+FTC-TP combination showed synergistic inhibition of HIV-1 RT in enzymatic assays

**Figure 4:**
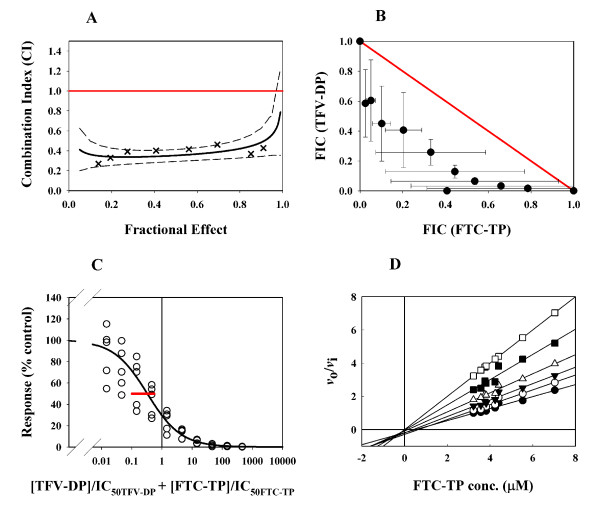
**Synergistic inhibition of HIV-1 RT by TFV-DP+FTC-TP combination analyzed by different methods**. (A) The median-effect analysis where the solid line presents curve fitting of the CI values as a function of the fractional effect. The red line at CI = 1 represents additivity. The dashed lines represent 95% confidence interval. TFV-DP and FTC-TP are combined at 1:1 IC_50 _ratios (1:10 molar ratios) with an average CI value of 0.47; (B) The isobologram analysis where the diagonally drawn solid line (in red) represents additivity. Average deviation from dose-wise additivity was -0.43 (*p *= 0.001), indicating a synergistic effect of the combination; (C) The Berenbaum combination indices analysis where the open circles are data from five experiments and the solid line represents the CI line. The red bar at the CI_50 _indicates the 95% confidence intervals. The bar is to the left of the CI_50 _= 1 line, indicating synergy for this combination. In this experiment, TFV-DP and FTC-TP were combined at 1:4 IC_50 _ratios (1:40 molar ratios); (D) The Yonetani-Theorell plot where FTC-TP was tested as the "first drug" and TFV-DP was added as the "second drug" at the following concentrations: (black circle) 0 μM, (open circle) 0.1 μM, (black triangle) 0.2 μM, (open triangle) 0.4 μM, (black square) 0.8 μM, and (open square) 1.6 μM. The Yonetani-Theorell plot shows the lines are converging at the left of the y-axis and the slopes of the lines increased with TFV-DP concentration, indicating the combination was synergistic.

An earlier study demonstrated a correlation between the synergistic antiviral effect of TFV+FTC combination, and the statistically significant increases in the levels of the active metabolites in T-cell line CEM[30]. To investigate whether the synergistic effect of TFV+FTC in cell culture could also be translated into synergistic inhibition at the enzymatic level, a standard HIV-1 RT inhibition assay was performed under steady state conditions using drug concentrations across the physiological range. In patients' peripheral blood mononuclear cells (PBMC) treated with TDF or FTC, the TFV-DP and FTC-TP concentration are 0.5 μM and 5 μM, respectively and are well within the range of the concentrations tested in the enzymatic assay [45,46]. The IC_50 _values for TFV-DP, FTC-TP and EFV were 0.53 ± 0.08, 5.0 ± 3.2, and 0.12 ± 0.01 μM, respectively when [α-^32^P]-dATP incorporation was used as the marker. The IC_50 _values for TFV-DP, FTC-TP and EFV were 0.82 ± 0.23, 2.4 ± 0.8, and 0.12 ± 0.01 μM, respectively, when [α-^32^P]-dCTP incorporation was used as the marker. The combination of TFV-DP+FTC-TP was first analyzed by the median-effect method. The combinations of TFV-DP+TFV-DP and FTC-TP+FTC-TP were tested as experimental controls, and as expected, they were additive regardless of whether ^32^P-dATP or ^32^P-dCTP was used as the radioactive marker in the assay (Table 2). The TFV-DP+FTC-TP combination was tested at three fixed IC_50 _ratios 1:3, 1:1, and 3:1, which corresponded to molar ratios of 1:30, 1:10, and 3:10, respectively. The results are summarized in Table 2. The combination of TFV-DP+FTC-TP was synergistic at all three IC_50 _ratios with CI values in the range of 0.47–0.61, regardless of whether ^32^P-dATP or ^32^P-dCTP was used in the assay. A representative median-effect analysis plot for the TFV-DP+FTC-TP combination is shown in Fig. 4A. In this experiment, TFV-DP and FTC-TP were combined at 1:1 IC_50 _ratio and the combination was tested at eight concentrations. The line at CI = 1 indicates the theoretical additive effect. It is evident that the combination of TFV-DP+FTC-TP had synergistic inhibitory effect on HIV-1 RT since the calculated CI for each of the eight drug combinations are well below 1.

**Table 2:** Combination Index (CI) values of TFV-DP, FTC-TP and EFV combination studies in the HIV-1 RT enzymatic assay.

Drug Combinations	^32^P-labeled dNTP	Ratio^a^	Average CI ± SD^b^	Degree of Synergy^c^
TFV-DP+TFV-DP	dATP	1:1	0.93 ± 0.07	additive

FTC-TP+FTC-TP	dATP	1:1	1.09 ± 0.28	additive
	
	dCTP	1:1	1.09 ± 0.25	additive

TFV-DP+FTC-TP	dATP	3:1	0.59 ± 0.04	synergy
		1:1	0.47 ± 0.09	synergy
		1:3	0.47 ± 0.04	synergy
	
	dCTP	3:1	0.60 ± 0.18	synergy
		1:1	0.61 ± 0.11	synergy
		1:3	0.47 ± 0.11	synergy

TFV-DP+EFV	dATP	3:1	0.69 ± 0.01	moderate synergy
		1:1	0.75 ± 0.04	moderate synergy
		1:3	0.94 ± 0.08	additive

FTC-TP+EFV	dATP	3:1	0.61 ± 0.08	synergy
		1:1	0.70 ± 0.03	moderate synergy
		1:3	0.81 ± 0.01	moderate synergy

TFV-TP+FTC-TP+EFV	dATP	3:3:1	0.37 ± 0.08	synergy
		1:1:1	0.49 ± 0.10	synergy
		1:1:3	0.67 ± 0.18	synergy

^a ^The ratio represents the biologically relevant IC_50 _ratios of the two compounds that were combined, based on their individual IC_50_s determined in the HIV-1 RT assay.

^b ^The averages and standard deviations were calculated from the results of at least three independent measurements.

^c ^Definition for degree of synergy is described in Methods.

To reduce the possibility of analysis bias, we further studied the combination of TFV-DP+FTC-TP using MacSynergy II analysis, which has been widely used to study drug combinations [29,30,47,48]. The TFV-DP+FTC-TP combination was found to be additive with a synergy/antagonism volume of 0.63/-2.7, which was calculated at the 95% confidence interval (Table 3). The discrepancy between the results from the median-effect analysis and the MacSynergy II analysis led us to analyze the combination using three other methods: the isobologram analysis, the Berebaum combination indices analysis with weighted non-linear regression, and the Yonetani-Theorell plots.

**Table 3:** Analyses of TFV-DP, FTC-TP and EFV combination studies in the HIV-1 RT enzymatic assay^a^.

Analysis Methods	Drug Combinations		
	
	TFV-DP+FTC-TP	TFV-DP+EFV	FTC-TP+EFV
Median-effect (CI value)	synergy^a^ (0.47 ± 0.09)	moderate synergy (0.75 ± 0.04)	moderate synergy (0.70 ± 0.03)

MacSynergy II^b^ (synergy/antagonism volume)	additive (0.63 ± 1.2/-2.7 ± 3.4)	minor synergy (44 ± 20/0 ± 0)	minor synergy (41 ± 15/-0.5 ± 1.1)

Isobologram (ADA^c^, *p *value)	synergy (-0.43, 0.001)	synergy (-0.20, 0.001)	synergy (-0.14, 0.002)

Berenbaum Combination Indices	synergy	ND^d^	ND

Yonetani-Theorell Plots	synergy	synergy	synergy

^a ^Definition for degree of synergy (whenever applicable) is described in Methods.

^b ^The volumes are calculated at the 95% confidence interval. The values are averages from 3 to 4 independent measurements. Five parallel repeats were tested in each measurement.

^c ^ADA = average deviation from additivity

^d ^ND = not determined.

In the representative isobologram plot of the TFV-DP+FTC-TP combination shown in Fig. 4B, the x-axis and y-axis represent fractional inhibitory concentration (FIC) of FTC-TP and TFV-DP, respectively. The calculated combination effects, shown by closed circles with bi-directional error bars calculated from five replicates, are all under the additivity line, indicating that the TFV-DP+FTC-TP combination is synergistic (ADA value of -0.31; *p *= 0.002). The TFV-DP+FTC-TP combination was further tested using analysis based on Berebaum Combination Indices (CI) with weighted non-linear regression to study the TFV-DP+FTC-TP combination. As shown in Fig. 4C, the red bar indicates the 95% confidence interval and its relative position to the CI_50 _= 1 line reveals the effect of combination. The bar is to the left of the CI_50 _line, suggesting synergy for the TFV-DP+FTC-TP combination (Table 3).

The Yonetani-Theorell plot was the method used by White *et al*. to conclude that combinations of AZT-TP+ddCTP, AZT-TP+ddATP, and AZT-TP+CBV-TP were all additive when tested for inhibition of HIV-1 RT, even though all these drug combinations were synergistic for inhibition of HIV-1 in cell culture studies [33]. In our study, we used this method to analyze the TFV-DP+FTC-TP combination (Fig. 4D). For a range of TFV-DP concentrations (0–1.6 μM), the reciprocal of the ratio of initial rate over *v *(*v*_0_/*v*) was plotted against the concentration of FTC-TP and the data were fitted with linear regression. Synergistic inhibition was observed for the TFV-DP+FTC-TP combination, as shown by the non-parallel and converging lines at the left of the y-axis (Table 3).

### TFV-DP+EFV combination showed synergistic inhibition of HIV-1 RT

To further understand the synergy of HIV-1 inhibition by TFV+EFV in cell culture, the combination was tested at the enzymatic level. The TFV-DP+EFV combination was tested by using ^32^P-dATP only since the TFV-DP+FTC-TP combination using ^32^P-dATP or ^32^P-dCTP showed nearly identical results. The combination of TFV-DP+EFV was tested at a 3:1, 1:1, and 1:3 IC_50 _ratios, which corresponded to 15:1, 5:1, and 5:3 molar ratios, respectively. As shown in Table 2, the combination of TFV-DP+EFV showed moderate synergy at 3:1 ratios (CI = 0.69) and 1:1 IC_50 _ratios (CI = 0.75), and additivity at 1:3 IC_50 _ratio (CI = 0.94). This combination was further tested using the three other methods (Table 3): the MacSynergy II analysis indicated that the combination showed minor synergy with a synergy/antagonism volume of (44/0); the isobologram analysis showed the combination to be synergistic with an ADA value of -0.20 (*p *= 0.001); and the Yonetani-Theorell plots of the combination demonstrated synergy (data not shown).

### FTC-TP+EFV combination showed synergistic inhibition of HIV-1 RT

To further understand the synergistic anti-HIV-1 effect of FTC+EFV, the combination was evaluated at the enzymatic level as well. The combination was tested using ^32^P-dATP at 3:1, 1:1, and 1:3 IC_50 _ratios, which correspond to 150:1, 50:1, and 50:3 molar ratios, respectively. As shown in Table 2, combination of FTC-TP+EFV showed synergy at the 3:1 IC_50 _ratio and moderate synergy at the 1:1 and 1:3 IC_50 _ratios, with CI values of 0.61, 0.70, and 0.81, respectively. This combination was further analyzed by three other methods (Table 3): the MacSynergy II analysis showed the combination showed minor synergy with a synergy/antagonism volume of (41/-0.5); the isobologram analysis showed the combination was synergistic with an ADA value of -0.14 (*p *= 0.002); and the Yonetani-Theorell plots of the combination demonstrated synergy (data not shown).

### Triple drug combination TFV-TP+FTC-TP+EFV showed synergistic inhibition of HIV-1 RT in enzymatic assays

The HIV-1 RT inhibitory effects of triple drug combination TFV-DP+FTC-TP+EFV were evaluated by the median-effect analysis. Earlier studies of the two drug combinations TFV-DP+FTC-TP at a 1:1 IC_50 _ratio showed synergy, therefore, TFV-DP: FTC-TP ratio was kept at a constant 1:1 IC_50 _ratio in this triple drug combination study. The combination was tested using ^32^P-dATP at 3:3:1, 1:1:1, and 1:1:3 IC_50 _ratios that correspond to 15:150:1, 5:50:1, and 5:50:3 molar ratios, respectively. This combination was synergistic at all three IC_50 _ratios tested with CI value ranges from 0.37–0.67 (Table 2).

### DEC formation by TFV-terminated DNA and FTC-MP-terminated DNA

The dead-end complex (DEC) refers to a salt-stable complex formed by HIV-1 RT/ddNMP-terminated DNA primer-template bound to the next dNTP (or ddNTP) that is resistant to being competed apart with excess template [49,50]. When DEC forms, HIV-1 RT and the DNA primer-template are "trapped" in a state where the forward reaction (polymerization), backward reaction (terminal ddNMP-excision), or enzyme-DNA dissociation cannot occur. We investigated the DEC formation using TFV-terminated DNA/RT/FTC-TP and FTC-MP-terminated DNA/RT/TFV-DP to test the hypothesis that the incoming NRTI might act as the next nucleotide to the chain-terminated primer and form a DEC, thus stabilizing the pre-existing chain-termination. Similarly, we speculated that DEC formation by TFV- or FTC-MP-terminated DNA with HIV-1 RT could be augmented in the presence of EFV, which could play an important role in the mechanism of action for the synergistic effects of TFV+EFV and FTC+EFV combinations observed in cell culture and in HIV-1 RT enzymatic assays. For these studies, formation of DEC was determined by three kinetic constants including the dissociation constant (*K*_d_), the maximum percentage of DNA primer-template forming a tight binding RT-DNA complex (B_max_), and the ratio of B_max_/*K*_d _which reflects the efficiency of DEC formation. Furthermore, two sets of DNA primer/templates (D20/D36 and D26/D50) were used to address whether the observation was sequence-dependent.

**Figure 5:**
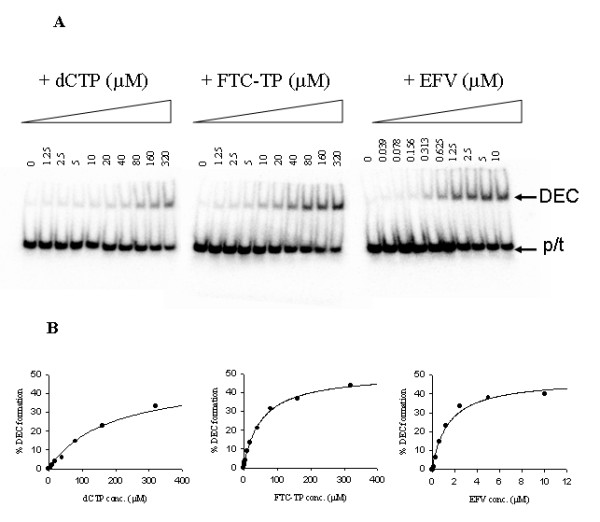
**DEC formation by HIV-1 RT, TFV-terminated DNA 26/50-mer**. (A) DEC formation by dCTP, FTC-TP, or EFV analyzed on a 6% non-denaturing polyacrylamide gel. (B) Quantification of the DEC formed by TFV-terminated DNA with HIV-1 RT in the presence of dCTP, FTC-TP, or EFV. Please note that the EFV x-axis is scaled significantly different from dCTP and FTC-TP. The amounts of free primer/template and DEC where quantified, and the percentage of DEC formed was plotted as a function of the concentration of dCTP, FTC-TP, or EFV. The solid line represents curve fitting of the data using the single ligand binding equation y = (B_max _× [ligand])/(*K*_d _+ [ligand]). The DEC formation by TFV-terminated DNA/RT in the presence of dCTP yielded B_max _= 49.2 ± 2.7% and *K*_d _= 185 ± 28 μM (left panel); The DEC formation by TFV-terminated DNA/RT in the presence of FTC-TP with B_max _= 50.4 ± 1.1% and *K*_d _= 52.6 ± 3.4 μM (middle panel); The DEC formation by TFV-terminated DNA/RT in the presence of EFV with B_max _= 48.0 ± 2.7% and *K*_d _= 1.43 ± 0.25 μM (right panel).

First, DEC formation using TFV-terminated DNA primer/template and HIV-1 RT was tested in the presence the next correct nucleotide dCTP or analogue FTC-TP. Along with TFV-terminated DNA, ddAMP-terminated DNA was also studied in parallel. As shown in Fig. 5a and Table 4, TFV-terminated DNA was able to form DEC with RT in the presence of dCTP or FTC-TP. Interestingly, TFV-terminated DNA+RT formed DEC with incoming FTC-TP as efficiently as with dCTP, but DEC formed with ddAMP-terminated DNA+RT and FTC-TP 10-fold less efficiently than with dCTP. Further observations showed that among the NRTI combinations, ddAMP-terminated DNA+RT in the presence of dCTP had the highest efficiency for DEC formation in the NRTI as the next nucleotide experiments (*B*_max_/*K*_d _= 1.6 for D20/D36 and 6.9 for D26/D50).

**Table 4:** Formation of DEC or DEC-like complex by ddNTP-terminated DNA and HIV-1 RT in the presence of next correct dNTP, ddNTP or EFV.

Dideoxyadenosine analog as the chain-terminator										
			Next Correct dNTP or ddNTP						EFV	
							
DNA	Terminator		dCTP			FTC-TP				
										
		*K*_d _(μM)	B_max _(%)	B_max_/*K*_d_	*K*_d _(μM)	B_max _(%)	B_max_/*K*_d_	*K*_d _(μM)	B_max _(%)	B_max_/*K*_d_

D20/D36	TFV	232 ± 12^a^	18.5 ± 6.7	0.079	250 ± 11	17.7 ± 3.9	0.071	0.70 ± 0.10	45.7 ± 10.0	65
	ddAMP	21.5 ± 9.8	34.2 ± 4.6	1.6	171 ± 28	28.1 ± 4.7	0.16	1.20 ± 0.38	16.3 ± 3.5	13.6

D26/D50	TFV	179 ± 33	50.0 ± 5.5	0.28	46.5 ± 7.8	50.0 ± 5.0	1.1	1.22 ± 0.28	44.4 ± 3.7	36
	ddAMP	8.1 ± 2.1	55.8 ± 3.4	6.9	86.8 ± 0.2	60.2 ± 7.2	0.69	2.42 ± 1.10	11.2 ± 0.6	4.6

Dideoxycytidine analog as the chain-terminator										

			Next Correct dNTP or ddNTP						EFV	
							
DNA	Terminator		dATP			TFV-DP				
										
		*K*_d _(μM)	B_max _(%)	B_max_/*K*_d_	*K*_d _(μM)	B_max _(%)	B_max_/*K*_d_	*K*_d _(μM)	B_max _(%)	B_max_/*K*_d_

D20/D36	FTC-MP	ND^a^	ND	ND	ND	ND	ND	1.54 ± 0.33	5.53 ± 1.83	3.0
	ddCMP	16.9 ± 8.5	38.0 ± 4.4	2.2	445 ± 114	15.3 ± 2.7	0.034	1.03 ± 0.25	14.9 ± 0.8	14.5

D27/D50	FTC-MP	862 ± 229	45 ± 14	0.052	ND	ND	ND	1.26 ± 0.24	3.96 ± 1.55	3.1
	ddCMP	6.5 ± 2.6	60 ± 3	9.2	140 ± 19	55 ± 2	0.39	0.11 ± 0.06	10.6 ± 2.0	96

All Values reported are average ± standard deviation from at least three measurements.

^a^ND = No DEC formation was detected at the highest concentration (2 mM) tested.

DEC formation using FTC-MP-terminated DNA primer/templates was studied in the presence of the next correct nucleotide dATP or analogue TFV-DP. As shown in Table 4, no DEC formation was detected for nearly all of the FTC-MP-terminated DNA with nucleotides, with the exception of the D27/D50-mer which showed low-level DEC formation in the presence of dATP, mainly caused by a very weak binding affinity (*K*_d _= 862 μM) of dATP to the RT-DNA complex. In contrast, ddCMP-terminated DNAs were shown to form DEC in the presence of dATP or TFV-DP, even though dATP induces 24- to 65-fold more efficient DEC formation than TFV-DP.

Surprisingly, EFV strongly promoted a stable complex that appeared biochemically DEC-like on all chain-terminated primers tested (Fig. 5 and Table 4), and did so more efficiently than with the natural dNTP, TFV-DP, or FTC-TP. In the presence of EFV, TFV-terminated DNA+RT and ddAMP-terminated DNA+RT formed stable complexes with efficiencies that were more than 820- and 8.5-fold higher than with dCTP, respectively. The TFV-terminated DNA+RT formed complexes with EFV 4.8- to 7.8-fold more efficiently than the ddAMP-terminated DNA+RT with EFV. In addition, appreciable levels of complex formation were detected for both FTC-MP and ddCMP-terminated DNA in the presence of EFV, with the FTC-MP-terminated DNA forming complexes 4.8- to 31-fold less efficiently than ddCMP-terminated DNA. Similar observations were found with both sets of DNA pair primer/templates, although the longer DNA D26/D50 was shown to be more efficient in forming complexes, which is in agreement with a previous report on DEC [51].

## Discussion

In this study, NRTI+NRTI and NRTI+NNRTI drug combinations were investigated using cell-based antiviral assays, HIV-1 RT enzymatic inhibition assays, and gel shift experiments. Even though many studies have shown the synergistic effect of NRTI combinations in cell culture, a few enzymatic studies suggested that these combinations inhibit HIV-1 RT additively [33,34]. These findings were readily accepted since it is conceptually hard to understand how two inhibitors that share the same mechanism of action could act synergistically. In 2006, our group reported the synergistic antiviral effect of TFV+FTC combination in cell culture studies and its correlation with elevated levels of the active metabolites of each drug [30].

In the current paper, we analyzed the TFV+FTC combination in cell culture using three methods including the median-effect, MacSynergy II, and the isobologram analysis, where the results showed synergistic effects, and were consistent with our earlier report [30]. The new studies include enzymatic experiments where five different analyses were used: the median-effect, MacSynergy II, isobologram, Berebaum combination indices, and the Yonetani-Theorell Plot analysis. Four of the five analyses showed that the combination of TFV-DP+FTC-TP led to synergistic inhibition of HIV-1 RT, with the exception of the MacSynergy II analysis, which showed the TFV-DP+FTC-TP combination to be additive. Interestingly, when the relevant subset of the same raw data which was found to be additive by the MacSynergy II analysis was re-analyzed with the median-effect analysis, isobologram analysis and the Yonetani-Theorell Plot analysis, all of the results showed the TFV-DP+FTC-TP combination to be synergistic. The discrepancy between the MacSynergy II and other analysis could be due to the fact that each method has its own statistical threshold, and the MacSynergy II analysis could have a more stringent criterion on calling whether the observed effect is synergistic. Whether the differences between the mathematic models of Bliss Independence and Loewe Additivity contribute to this discrepancy is beyond the scope of this study.

The molecular mechanism of action for the synergistic effects at the enzymatic level by two NRTIs remained unknown. NRTIs with differing base moieties should chain-terminate different sequences, but this would not explain synergy at the enzymatic level. Based on the reports that DEC can be formed by the assembly of 3'-terminated DNA, HIV-1 RT, and the next incoming natural dNTP or ddNTP [49-52], we hypothesize these NRTIs can sequester the viral DNA and HIV-1 RT in this inactive form, protect the NRTI-terminator from excision, and therefore enhance the inhibition of viral DNA synthesis introduced initially by the terminating NRTI. Our results showed that, using two different sequence contexts, FTC-TP could form DEC with TFV-terminated DNA+RT at levels that are equal to or 3.9-fold more efficient than dCTP. The relatively high intracellular concentration of FTC-TP (5 μM) in patients [46] relative to dCTP (0.7–23.2 μM) [53,54] suggests that our *in vitro *study is physiologically plausible. Our results are consistent with a recent report on the formation of DEC by TFV-terminated DNA+RT+dCTP, even though our reported *K*_d _values (179 μM) are notably higher than the 1–5 μM reported values [51,55]. However, our results are more in line with other reported values [50,52]. This discrepancy could be due to primer/template sequence effects which are known to significantly affect the *K*_d _values, different salt concentrations (60–160 mM KCl), and different times of incubation (15 min to 60 min) applied in each assay. Similar to a report on the lack of DEC formation by a 3TC-MP-terminated primer [50], we observed low-level DEC formation by FTC-MP-terminated DNA with dATP and no DEC formation with TFV-DP.

The observation that the ddAMP-terminated DNA+RT formed DEC more efficiently than the TFV-terminated DNA+RT with a dNTP or NRTI as the incoming nucleotide is consistent with the favorable binding of natural dNTP over ddNTP. Site-specific footprinting experiments in low dNTP concentrations showed that ddAMP-terminated primers existed predominantly in the post-translocational state which favors DEC formation, while TFV-terminated primers existed equally in both pre- and posttranslocational states and formed a DEC less favorably than ddAMP [55]. A similar trend was observed by Tong *et al*. where the preference of natural dNTP over ddNTP for DEC formation was reported [51].

An unexpected observation was that TFV-terminated DNA and FTC-TP formed DEC as efficiently or 4-fold *higher *than with dCTP. Structurally, the smaller and more flexible TFV at the primer terminus, driven by the absence of a cyclic sugar, might better accommodate the binding of FTC-TP compared to a ddAMP-terminated primer. It may also be possible that the terminal-TFV and incoming FTC-TP may adopt a conformation that more strongly favors the posttranslocational state. There are no reported studies on the translocation state of FTC-MP-terminated primers, but it is conceivable that the L-conformation of FTC severely disfavors the translocation of FTC-MP-terminated primer to the posttranslocational state and thereby precludes the formation of DEC. Our experimental data showed that FTC-MP-terminated DNA only formed low-levels of DEC in the presence of dATP, and no DEC in the presence of TFV-DP, while ddCMP-terminated DNA formed DEC with dATP or TFV-DP.

Combinations of NRTI+NRTI at the enzymatic level should be studied with a system carefully designed to be heterogeneous enough to reflect the "independent" incorporation of each single NRTI. In our assay, the activated calf thymus DNA was used instead of a DNA primer/template with defined sequence that might pre-condition the order of incorporation and bias the outcome [33,34]. It is noteworthy that TFV-DP could also induce a DEC with TFV-MP-terminated DNA/RT provided dATP is the next correct dNTP; however, this effect on "enhancing" RT inhibition is inseparable from the enzyme inhibition caused by chain-termination alone. In other words, an added effect (synergy) will not be observed when TFV-DP is added to itself.

A second focus of this paper analyzes the NRTI+NNRTI (two drug class) combinations of TFV+EFV and FTC+EFV using three different methods of analysis: the median-effect, the MacSynergy II, and the isobologram. All of these analyses showed that the TFV+EFV and FTC+EFV combinations synergistically inhibited HIV-1 replication. Furthermore, it was demonstrated that the combination of TFV-DP+EFV and FTC-TP+EFV are synergistic at the enzymatic level, and that both TFV-terminated and FTC-MP-terminated DNA formed stable, DEC-like complexes with HIV-1 RT in the presence of EFV. The *K*_d _values for EFV binding for stable complex formation was around 1 μM, which is well below the 5 μM steady-state plasma EFV concentration [56]. Our findings are consistent with the report by Cruchaga *et al*. on the formation of a stable complex by HIV-1 RT, AZT-terminated template-primer and EFV [19].

Based on our current study and the previously reported findings, we propose that there are at least three independent factors may contribute to synergistic effects of NRTIs and NNRTIs: (1) diminished ATP binding in the presence of NNRTIs decreases efficiency of excision for NRTI terminator [18,20]; (2) increased RNase H activity in the presence of NNRTIs can diminish opportunities for NRTI excision [21]; and (3) as shown in our work, NNRTI-mediated stable complex formation prolongs and enhances the chain-termination effects of NRTIs.

Remarkably, EFV induces stable complexes substantially more efficiently than the next base-paring dNTP or NRTI-TP (or -DP). Complexes formed with EFV may be far less sensitive to the translocational state of the chain-terminating NRTI than traditional DEC formed with dNTP or ddNTP. Furthermore, EFV formed complexes independently of the sequence of the next correct base, which restricts DEC formation by a nucleotide. The EFV-based complexes should be structurally distinct from the DEC formed by an incoming nucleotide. Additional insight on the structure of this complex was recently put forth by Abbondanzieri *et al*., where HIV-1 RT bound to chain-terminated polypurine track DNA in the presence of the NNRTI nevirapine (or EFV) showed increased RT/DNA association/binding time and "flipping" between polymerase- and RNase H-binding orientations, where the RNase-H/non-polymerase binding orientation was favored [57]. This nevirapine-based complex therefore showed a distinct pattern of binding orientations compared to a traditional DEC bound with dNTP where only the polymerase-competent binding orientation is observed. In either binding orientation, the EFV-based complex appears to be a DEC-like complex that is stable in the presence of high salt concentration and competing DNA, where the chain-terminated primer terminus presumably lies inaccessible to excision. Grobler *et al. *showed that NNRTIs potently and specifically inhibit plus-strand initiation and proposed that part of the NNRTI inhibition on HIV-1 RT-catalyzed polymerization is the result of trapping the enzyme in a polymerase-independent RNase H-competent mode of binding [58]. Our study demonstrated that part of the NNRTI inhibition may also come from the trapping of enzyme once the primer is terminated with a NRTI.

It is worth noting that even though EFV promoted DEC formation at a much higher level than TFV-DP or FTC-TP, drug combinations involving EFV did not yield a higher degree of synergy than the combination of TFV-TP+FTC-TP. It is possible that additional factors may come into play.

## Conclusion

In summary, the combinations of TFV+FTC, TFV+EFV, FTC+EFV, and TFV+FTC+EFV all showed synergistic anti-HIV activity in cell culture and synergistic inhibition of HIV-1 RT under steady state enzymatic kinetic conditions. Gel shift experiments suggest the efficient formation of DEC of RT/TFV-terminated DNA/FTC-TP, and DEC-like complexes of RT/TFV-terminated DNA/EFV and RT/FTC-MP-terminated DNA/EFV. We propose the following mechanisms contributing to the TFV+FTC+EFV synergy: (1) TFV+FTC combination results in increased levels of the active metabolites TFV-DP and FTC-TP [30]; and (2) DEC or DEC-like complex formation by TFV-terminated DNA and HIV-1 RT in the presence of the FTC-TP or EFV, and by FTC-MP-terminated DNA and HIV-1 RT in the presence of EFV. This study furthers our understanding of the mechanism of action for anti-HIV drug interactions and the efficacy observed for the TDF+FTC+EFV triple combination for the treatment of HIV infection.

## Methods

### Reagents

TFV, FTC, and EFV were synthesized by Gilead Sciences. All four natural dNTPs, ddATP, ddCTP, and [α-^32^P]dATP, [α-^32^P]dCTP, and [γ-^32^P]ATP were from GE Healthcare BioSciences (Piscataway, NJ). TFV diphosphate (TFV-DP) and FTC triphosphate (FTC-TP) were synthesized by ChemCyte, Inc. (San Diego, California, USA) as lithium salt, was > 95% pure by HPLC, and free of inorganic phosphate confirmed by ^31^P-NMR. Activated calf thymus DNA and poly(rA).poly(dT)_12–18 _were purchased from GE Healthcare BioSciences (Piscataway, New Jersey, USA). DNA oligonucleotide primers D19 (5'-GTCCCTGTTCGGGCGCCAC), D25 (5'-CTGAGACAACATCTGCTGAGGTAGG), and D26 (5'-CTGAGACAACATCTGCTGAGGTAGGA), and templates D36A (3'-CGAAAGTCCAGGGACAAGCCCGCGGTG TGTATCTCT), D36C (3'-CGAAAGTCCAGGGACAAGCCCGCGGTG GTAATCTCT), and D50 (3'-GACTCTGTTGTAGACGACTCCATCC TGTATGGTGTGCTGTGGTGTGCTGT) were prepared and PAGE purified by Integrated DNA Technologies, Inc. (Coralville, Iowa, USA). The underline base indicates the template base for the incoming dNTP. Concentrations of oligonucleotides were determined from the absorbance at 260 nm. XTT {(2,3-bis (2-methoxy-4-nitro-5-sulfophenyl)-5-[(phenylamino) carbonyl]-2H-tetrazolium hydroxide} was from Sigma-Aldrich (St. Louis, Missouri, USA).

### Recombinant HIV-1 RT enzyme construction and purification

Wild-type HIV-1 RT p66/p51 heterodimers containing N-terminal 6-His tags were cloned and purified as previously described [3]. The active site concentration was 52% for HIV-1 RT determined by pre-steady state kinetic analysis [59].

### Antiviral combination assays

Human T leukemia MT-2 cells were obtained from the NIH AIDS Research & Reference Reagent Program and were maintained in RPMI 1640 media supplemented with 10% FBS, 50 μg/mL gentamicin and 0.29 μg/mL glutamine. MT-2 cells were infected with HIV-1 strain xxLAI [60] at a multiplicity of infection of 0.03 for 3 hrs, washed once with complete media, and plated at a final concentration of 3 x10^4 ^cells/well in 96-well plates containing various concentrations of test compound. The infected cells were incubated for 5 days at 37°C in 5% CO_2_. Antiviral activity was measured by determining the HIV-1 cytopathic effect by using the vital dye XTT (2,3-bis(2-methoxy-4-nitro-5-sulphophenyl)-2H-tetrazolium-5-carboxanilide) based colorimetric assay [61]. TFV, FTC, and EFV were first tested individually for effective concentrations which inhibited 50% of viral replication (EC_50_) using SigmaPlot 9.0 (Systat Software Inc., San Jose, CA).

For the median-effect analysis, the compounds were combined at a 1:1 ratio based on their EC_50_. Six to eight concentrations of each single drug, two-drug combinations, and three-drug combinations were assayed in at least three independent experiments with quadruplicate wells for each experiment. The triple drug combination of TFV+FTC+EFV was studied by the median-effect analysis only. The triple drug combination of TFV+FTC+EFV was studied by the median-effect analysis only. For the MacSynergy analysis, combinations of TFV+FTC, TFV+EFV, and FTC+EFV were tested in a checkerboard fashion in 96-well plates with the starting concentration for each compound fixed at three-fold to four-fold above the EC_50 _value. For the isobologram analysis, combinations of TFV+FTC, TFV+EFV, and FTC+EFV were tested in a checkerboard fashion with the starting concentration for each compound fixed at three-fold to four-fold above the EC_50 _value.

### HIV-1 RT enzymatic combination assay

All concentrations listed were final except noted otherwise. All RT reactions were conducted as follows: A solution (buffer A) containing 50 mM Tris-HCl, 5 mM MgCl_2_, 60 mM KCl, 5 mM DTT, 4.2% (v/v) glycerol, 300 μg/mL bovine serum albumin, 400 nM dATP, 400 nM dCTP, [α-^32^P]dATP (or [α-^32^P]dCTP, 0.03 μCi/μL), various concentrations of each single inhibitor (or their combinations) and 5 nM HIV-1 RT was pre-incubated at 37°C for 5 min. The reaction was initiated by mixing buffer A with a pre-incubated mixture (buffer B) of 50 μM dGTP, 50 μM TTP, and 3 mAU/μL activated calf thymus DNA. The 75 μL reaction mixture was incubated at 37°C and 5 μL aliquots were removed and spotted onto Whatman DE81 anion exchange paper at 1, 1.5, 2, 2.5, and 3 min time interval (for fixed-ratio setup [62] or at a single time point 3 min (for checkerboard setup [42]. After washing with Na_2_HPO_4 _buffer (50 g/L, 3 × 5 min), distilled water (5 min) and ethanol (5 min), the paper was air-dried and exposed to a phosphor storage screen. Product formation was quantified using GE Storm 820 PhosphorImager and ImageQuant TL software (GE Healthcare, Piscataway, New Jersey, USA). The observed rate for the HIV-1 catalyzed reaction was quantified by linear regression of the product formed as a function of time (Kaleidagraph, 4.0, Synergy Software, Reading, Pennsylvannia, USA). Less than 2% substrate was consumed in the time frame tested. For each inhibitor concentration, percentage inhibition was calculated by using no-drug as 0% inhibition. The IC_50 _values were calculated using Sigma Plot 9.0 (Systat Software, San Jose, California, USA).

### Drug combination data analysis

#### Median-effect analysis

The degrees of synergy and antagonism were determined using CalcuSyn software (version 2.0, Biosoft, Cambridge, UK) which is based on median-effect principle by Chou and Talalay [62]. In this analysis, the log (*f*_a_/*f*_u_) is plotted as a function of the log [*I*], where *f*_a _is the fractional inhibition caused by the drug relative to the no drug control, *f*u is the fractional uninhibited level (1-*f*_a_), and [*I*] is the drug concentration. The IC_50 _value is calculated from the y-intercept (-m * *log *IC_50_), where *m *is the slope of the curve. The following equation represents how the combination index (CI) is defined in a mutually exclusive model:



Where 1 and 2 represent the individual action of drug 1 or drug 2, while 1,2 represents the combined action of the drug combination. The CI values of < 1, = 1, and > 1 indicate synergy, additive effect, and antagonism, respectively. The degree of synergy is categorized based on the CI value: very strong synergy (< 0.1), strong synergy (0.1 to 0.3), synergy (0.3 to 0.7); moderate synergy (0.7 to 0.85), additive (0.85 to 1.20), moderate antagonism (1.20 to 1.45), antagonism (1.45 to 3.3), strong antagonism (3.3 to 10), and very strong antagonism (> 10). The 95% confidence interval at each level of fractional inhibition (*f*_a_) is calculated by the formula: CI ± (1.96 × S.D.), where the values CI + (1.96 × S.D.) and CI - (1.96 × S.D.) correspond to the upper and lower boundaries of the confidence interval, respectively.

All drug combinations were tested at increasing total drug concentrations that were set at fixed 1:1 ratios based on the single drug IC_50 _values for cell-based combination assays. For HIV-1 RT enzymatic assays, two drug combinations were tested using three series of experiments with increasing total drug concentrations that remained fixed at IC_50 _ratios of 1:3, 1:1, and 3:1, while the three drug combination TFV-DP+FTC-TP+EFV experiments were tested at 3:3:1, 1:1:1, and 1:1:3 fixed IC_50 _ratios. Each drug ratio experiment was repeated at least three times and for each experiment a set of CI values was reported at 50%, 75%, 90% and 95% (EC_50_, EC_75_, EC_90_, EC_95_) inhibition levels. In many cases, the CI value decreased as the fractional inhibition (*f*_a_) increased, as commonly seen in many synergistic combinations. Therefore, the averaged CI values at EC_50_, EC_75_, EC_90_, and EC_95 _were used as recommended by the program manual. In some occasions when the effect of one single drug did not reach the 95% inhibition level, the EC_95 _value for the combination was not included for the average CI calculation. Data points that reach > 99% inhibition were excluded from the analysis.

#### MacSynergy II analysis

The MacSynergy II program (version 1.0, Ann Arbor, MI) calculates a theoretical additive value for each drug combination based on the values generated by the drugs alone using the Bliss Independence model [42]. The theoretical additive values are subtracted from the experimental values generated by each drug combination to give a value of synergy (positive value) or antagonism (negative value). These synergy and/or antagonism values are plotted on a three-dimensional graph with their corresponding drug combinations. Areas of the graph blow zero indicate antagonism, whereas areas above zero indicate synergy. A synergy volume is calculated by adding all of the positive values for each drug combination. Similarly, all of the negative values are added to give an antagonistic volume. These synergy and antagonism volumes are then statistically evaluated using the 95% confidence level and are expressed in μM^2^%, which are used to categorize the degree of synergy: strong synergy (>100), moderate synergy (50 to 100), minor synergy (25 to 50), additive (-25 to 25), minor antagonism (-25 to -50), moderate antagonism (-50 to -100), and strong antagonism (< -100) [63].

In our cell culture study, the score of synergy/antagonism was calculated from a set of 5 replicate parallel measurements and more than 3 sets of such experiments were conducted to test the reproducibility of the results. Compounds were tested in a checkerboard fashion in 96-well plates with the starting concentration for each compound fixed at threefold to fourfold above the EC_50 _value. Compound #1 was tested at 8 concentrations with 2-fold serial dilutions down the plate, while compound #2 was tested at 12-concentrations with 2-fold serial dilutions across the plate. The concentration range of the individual drugs was carefully selected to ensure that the inhibition by each drug remained <95% at the highest concentration. It is known that when single drug reaches >95% inhibition, any additive or synergistic effect of drug combination can no longer be detected and the effect is often scored as antagonism [63].

The enzymatic study was conducted in a similar manner, where synergy was calculated from a set of 5 parallel measurements and 3–4 independent sets of experimental data were collected. The starting concentration for each compound was fixed at 3–4 fold above the individual drug IC_50 _value.

#### Isobologram analysis

The isobologram analysis is a graphical approach that can be traced back to as early as the eighteen century [35], and is time-tested and widely accepted [40]. The combination studies are generally conducted using a checkerboard setup where dose-response curves are generated for each drug alone and in combination and used to determine EC_50 _values for each drug alone or in the presence of the fixed concentration of the second drug using GraphPad Prism (version 4.0, Systat Software Inc., San Jose, California, USA). Fractional inhibitory concentrations are calculated by dividing the EC_50 _of drug 1 with a fixed overlay of drug 2 by the EC_50 _of drug 1 alone (the x-coordinate). The y-coordinate is the fixed concentration of drug 2 divided by the EC_50 _of drug 2 alone. These points are plotted on a graph to generate the isobologram. On the same graph, a diagonally drawn line represents "additivity" by linking coordinates (1, 0) to (0, 1) (Fig 3B). Data points that are above the additivity line represent antagonism between the compounds whereas data points below the additivity line represent synergy between the compounds. The intensity of the interaction can be measured by the statistical difference from dose-wise additivity tested by a one-tailed *t*-test [41]. The average deviation from additive (ADA) is reported with a *p *value. A negative ADA value indicates synergy and a value of -0.5 indicates strong synergy, however there isn't a clearly defined scoring system for synergy analyzed by quantitative isobologram [41]; therefore, we defined combinations with ADA values between 0 to -0.5 to be synergistic, as long as the values were statistically significant (*p *< 0.05).

#### Berenbaum combination indices approach

The Berenbaum combination (or interaction) indices method [64] is mathematically identical to the isobologram analysis described earlier. The experimental condition is very similar to the ones used in fixed ratio assays, but a single 5-minute time point was collected for each reaction due to the requirement of large data collection. The product formation at 5 min is within the linear range of the assay. A total of 5 different fixed-ratio combinations of the two drugs (1:1, 1:2, 2:1, 1:4, and 4:1 EC_50 _ratio), plus each agent alone, and 88 design points in quintuplicate for a total of 440 data points were used in the modeling. The analysis started with modeling of the error structure and calculation of variance and means for each of the 88 sets of 5 replicates and derived weighting factor for subsequent nonlinear regression runs. Next, the data for each single agent and the five different fixed-ratio combinations were fitted to the four parameter Hill equation (see below) with transformed x-axis (C_1_/IC_50 _+C_2_/IC_50_) with iteratively reweighed nonlinear regression using SAS v. 9.1 NLIN. The value represented by C_1_/IC_50 _+ C_2_/IC_50 _is CI_50_, which is equal to the combination indices at the IC_50 _level and is a measure of synergy (CI_50 _< 1), or additivity (CI_50 _= 1), or antagonism (CI_50 _> 1). As shown in Figure 3C, the red bar indicates the 95% confidence interval and its relative position to the CI_50 _= 1 line reveals the effect of combination. When the bar is to the left of the CI_50 _line, synergy is indicated; when the bar is to the right, antagonism is indicated; and when the bar crosses the CI_50 _= 1 line, additivity is indicated.



Where E is the output (effect or response), and C is the input (concentration of agent). The E_con _parameter is the level of measured effect at zero drug concentration; the B background parameter is the level of measured effect at infinite drug concentration; and m is called the Hill sigmoidicity or slope parameter.

#### Yonetani-Theorell plot

The Yonetani-Theorell plot approach has been used by many groups in the past to study antiretroviral drug combinations [33,65-67]. Even though it was a popular choice in the 1980's-1990's, Yonetani-Theorell Plot is known today for oversimplifying and misinterpretation for certain drug combination studies [68]. However, this method was used in our study to provide a way to compare our analysis with previous published studies where the Yonetani-Theorell plot analysis was the only method employed. In the absence of the second drug, the reciprocal of the ratio of the initial rate in the absence of inhibitors over v (v_0_/v) is plotted against the concentration of first drug and the data are fitted with linear regression [43]. A set of such lines was then generated for the first drug with increasing concentrations of the second drug. Synergistic inhibition is detected by lines converging at the left of the y-axis, while parallel lines indicate additivity. Diverging lines (lines crossing at the right of the y-axis) indicate antagonism [19].

### DEC formation assay

DNA primers D19, D25, and D26 were 5'-end labeled with T4 polynucleotide kinase (New England Biolabs, Ipswich, MA) and [γ-^32^P]ATP as previously described [69]. All DNA/DNA primer/templates were annealed by incubating a 1:1.3 molar ratio of primer to template in 50 mM Tris-HCl buffer containing 50 mM NaCl at 90°C for 3 min, 50°C for 10 min, and left on ice for 10 min. The annealed primer/templates were analyzed by non-denaturing polyacrylamide gel (4–20% TBE) electrophoresis to ensure that the proper annealing had taken place.

The ability of HIV-1 RT to form a stable complex with NRTI-terminated primer/template in the presence of dNTP, ddNTP or NNRTI was assessed as previously described with modification [19,50,51]. Two sets of primer/templates were tested using D19/D36A (D19/D36C) and D25/D50 (D26/D50). Labeled primer/template was chain-terminated by incubating a mixture containing 50 mM Tris-HCl (pH 7.8), 60 mM KCl, 5 mM MgCl_2_, 5 mM DTT, 300 μg/mL bovine serum albumin, 4.2 (v/v) % glycerol, 5 nM [5'-^32^P]-labeled primer/template, 0.5 μM of ddATP or TFV-DP (for D19/D36A or D25/D50) or 0.5 μM of ddCTP or FTC-TP (for D19/D36C or D26/D50), and 200 nM HIV-1 RT for 5 min at 37°C and then placed on ice. A fraction of the reaction mixture was saved and later analyzed by sequencing gel electrophoresis (16% polyacrylamide, 8 M urea). All of the primers tested were shown to be > 95% extended to n+1 product. Free dNTP, ddNTP, or EFV was added into the mixture and the mixture was incubated for 15 min at 25°C. At the end of the incubation, the mixture was placed on ice and mixed with 100 mM KCl and 1 A_260 _unit/mL (for D19/D36A and D19/D36C) or 5 A_260 _unit/mL (for D25/D50 and D26/D50) poly(rA).poly(dT)_12–18_. Control studies showed that DEC is intact in the presence of high salt 100 mM KCl, while regular binary RT/primer-template complexes dissociate and RT is trapped by cold poly (rA).poly (dT)_12–18 _and is prevented from rebinding to ^32^P-labeled DNA. After incubation at 37°C for 5 min, the reaction mixture was cooled on ice, mixed with native loading dye (30% glycerol, 0.25% bromophenol blue) and analyzed on a 6% non-denaturing polyacrylamide gel in 0.5 × TBE buffer (0.45 M Tris-borate, 0.01 M EDTA, pH 8.3) on ice for 1 h (100 volts). The gel was exposed to a storage phosphor screen and DEC formation was quantified using GE Storm 820 PhosphorImager and ImageQuant TL software (GE Healthcare, Piscataway, NJ).

The DEC formation was analyzed by plotting the fraction of the primer/template detected as a function of dNTP, ddNTP, or EFV concentration. Examples of dCTP, FTC-TP, and EFV-facilitated DEC formation and the data fittings are illustrated in Fig 5. Data were fitted by nonlinear regression to simple ligand binding equation: y = (B_max _× [ligand])/(*K*_d _+ [ligand]) using SigmaPlot 9.0 (Systat Software, Inc., San Jose, CA), where the ligand is the free dNTP, ddNTP, or EFV concentration, and y corresponds to the % of primer/template in DEC complex. The kinetic constants *K*_d _represents the apparent dissociation constant of a ligand and B_max _is the maximum % of DEC complex formation.

## Abbreviations

ABC: abacavir; ADA: the average deviation from additivity; AZT: 3'-azidothymidine; DEC: dead-end complex; DP: 5'-diphosphate; EFV: efavirenz; FTC: emtricitabine; PBMC: peripheral blood mononuclear cells; RT: reverse transcriptase; NVP: nevirapine; NNRTI: non-nucleoside reverse transcriptase inhibitor; NRTI: nucleoside or nucleotide reverse transcriptase inhibitor; TFV: tenofovir; TDF: tenofovir disoproxil fumarate; TP: 5'-triphosphate; XTT: 2,3-bis(2-methoxy-4-nitro-5-sulphophenyl)-2H-tetrazolium-5-carboxanilide.

## Competing interests

All of the authors are full-time employees of Gilead Sciences, Inc. and shareholders of the company.

## Authors' contributions

JYF designed and conducted the biochemical assays and drafted the manuscript. JKL carried out a portion of the biochemical assays. FM and DG carried out the cell-based drug combination assays. KLW and ESS participated in the study design, data analysis, and manuscript preparation. KBE and MDM participated in the preparation of the manuscript.
